# Does Erythropoietin Cause Hemoglobin Variability- Is It ‘Normal’?

**DOI:** 10.1371/journal.pone.0092890

**Published:** 2014-04-07

**Authors:** Ashwani K Gupta, Waseem David

**Affiliations:** Department of Nephrology, University of Florida-Jacksonville, Jacksonville, Florida, United States of America; French Blood Institute, France

## Abstract

Hemoglobin variability (Hb-var) in patients with chronic kidney disease has been stipulated to be a result of exogenous treatment with erythropoiesis stimulating agents (ESA) and has been related to mortality in dialysis patients. We hypothesized the existence of Hb-var independent of ESA administration and compared it to that in healthy adults using data from the Scripps-Kaiser and NHANES III databases. We studied the Hb-var in 1571 peritoneal dialysis patients which included 116 patients not requiring treatment with erythropoietin. We systematically studied the differences between the groups that needed ESA therapy and those who did not. White race and male sex were significant predictors of need for erythropoietin therapy. We found peritoneal dialysis patients to exhibit significantly increased Hb-var independent of treatment with exogenous erythropoietin (0.99 gm/dL vs. 1.17 gm/dL, p-value<0.001). We found age to be a significant determinant of Hb-var in the ESA treated group. Hb-var in younger patients (<30 years) was increased by 50% compared to young healthy adults. The Hb-var in elderly (>60 years) peritoneal dialysis patients was similar to that seen in healthy elders, suggesting similarity with anemia of aging. We conclude that exogenous ESA administration does not explain Hb-var entirely but may enhance it. Intrinsic factors affecting erythropoiesis including age may be the major determinants of Hb-var.

## Introduction

The basal rate of Red Blood Cell (RBC) production is diminished in patients with chronic kidney disease (CKD). Usually, the anemia is proportional to the severity of renal dysfunction, caused due to a deficiency of erythropoietin (EPO) [1]. Supplementation with exogenous Erythropoiesis Stimulating Agents (ESA) partially corrects the EPO deficiency and is used for its treatment [2]. Treatment of anemia in this population has several benefits including improvement in exercise tolerance, functional and sexual capacity [3], along with reduction in left ventricular hypertrophy [4], and improved cardiovascular functioning. A large variability has been observed in the hemoglobin level of dialysis patients treated with ESAs [5]. The physiologic basis underlying hemoglobin variability (Hb-var) is poorly understood. It is thought that patient demographics, comorbidities, concurrent events (infections and hospitalizations) as well practice patterns of ESA and iron administration are related to Hb-var. Several investigations [6]–[11] have focused exclusively on the effects of ESA on Hb-var in hemodialysis patients. This phenomenon has been called Hb cycling [5], [12] by clinical epidemiologists and has been related to mortality [13]–[16] and cardiovascular outcomes.

Data on Hb-var in peritoneal dialysis (PD) patients are limited to small reports from the Netherlands [17], the United Kingdom [18] and China [19]. Walker et al. [20] have reported the largest group of 558 PD patients receiving Erythropoietin and 528 PD patients receiving Darbepoetin from the Australian continent. They observed an Hb standard deviation of 1.5 gm/dL in the Darbepoetin group, as compared to 1.1 gm/dL in the erythropoietin treated group. No data have been reported from the United States or Canada. A large cohort of European dialysis patients [21] included data on 967 patients not receiving ESA, but the authors did not analyze this data separately. We hypothesize the existence of Hb-var in PD patients not receiving any ESA and that the Hb-var in PD patients would be comparable to or greater than healthy adults. This has not been reported in the literature previously. In this study, we examined Hb-var data on 1571 PD patients who received dialysis at various Dialysis Clinics Inc. (a large non-profit dialysis provider) facilities throughout the United States between January 2007 and December 2009. The study attempts to define “normal” Hb-var in CKD by comparing our results with population normal in healthy adults obtained from the Scripps-Kaiser and NHANESIII databases [22]. In order to determine the relationship of Hb variability to ESA administration, we compared the subset of PD patients not receiving any ESA to the group receiving ESAs. The study of the subset of CKD patients not receiving ESA is a natural first step in understanding the red blood cell dynamics in renal failure. We will attempt to describe how this subset of patients differs in characteristics from those who require ESA therapy. We will further attempt to investigate the factors that determine Hb variability in both subsets of patients.

## Methods

### Study Design

This was a retrospective observational study. The study protocol was approved by the University of Florida Institutional Review Board and adhered to the declaration of Helsinki. A limited data set consisting of de-identified data was provided by Dialysis Clinics Inc. through a data use agreement. It was not possible to identify individual patients directly or through identifiers linked to the patients. Therefore, it was exempted from requiring informed consent by the IRB. Prevalent peritoneal dialysis patients receiving services for 6 months or longer at any of the centers operated by Dialysis Clinic Inc., a large nonprofit dialysis provider in the United States between January 2007 and December 2009 were included in this study. Children less than 18 years of age were excluded from the study population. Demographic data including - age, sex, race, dialysis vintage were included in the data provided. Date of clinic visit, Hb measured per clinic protocol (monthly); ESA dose administered, types of ESA used were also recorded. Laboratory values obtained during routine clinical care including serum iron, ferritin, TIBC, percent saturation (usually every 3 months), serum Albumin, PTH, and Kt/V measured per clinic protocol were recorded.

### Data Analysis

All data analysis was carried out using STATA v.9.0. Patients were stratified into those requiring any ESA therapy (n = 1455) and those not requiring ESA therapy (n = 116). Patients who were administered even a single dose of an ESA during the study period were stratified in the ESA group. ESA naïve patients did not receive any doses of an ESA during the entire follow-up period. Hb-var was calculated using a 1 month standard deviation (SD-Hb) and the coefficient of hemoglobin variation. The coefficient of hemoglobin variation is defined as the ratio of the SD-Hb to the mean Hb. Results are reported as mean ± standard deviation. Comparisons between groups were based on a two sided student's t-test of means. The patients were divided into deciles of age to compare Hb-var with the population means reported in the Scripps-Kaiser and NHANESIII databases [22]. A regression model was fit to SD-Hb in the ESA treated group to study the effect of various parameters on SD-Hb.

## Results

Data was available for a total of 1571 patients followed over a mean 19 months (range 7–156 weeks). The data revealed that 7.4% (n = 116) of our sample did not require any ESA therapy over the entire follow-up period. The two groups did not differ with regards to mean patient age, dialysis vintage and follow up period in the study. The proportion of diabetics in either group was also similar. Table 1 shows the differences between the no-ESA vs. ESA group. As expected, the mean Hb in the no-ESA group was 13.0 explaining the lack of need for ESA therapy. The ESA group was treated to a mean Hb of 11.7. The standard deviation of hemoglobin in the ESA group was 1.17 gm/dL compared to 0.99 gm/dL in the no-ESA group (p-value<0.001).

**Table 1:** Differences between ESA treated and ESA Naïve PD Patients.

	No-ESA			ESA			P-value
Variable	Obs(n)	Mean	Std. Dev.	Obs(n)	Mean	Std. Dev.	
Mean-Hb(gm/dL)	116	13.0	1.7	1455	11.7	0.9	<0.0001
SD-Hb*(gm/dL)	116	0.99	0.45	1455	1.17	0.43	<0.0001
CV-Hb#	116	7.70	3.60	1455	10.00	3.80	<0.0001
Age(years)	116	59.9	13.37	1455	59.9	14.25	0.9
Sex							
Male	80	69%		773	53%		0.004
Female	36	31%		682	47%		
Vintage(years)	114	6.3	2.8	1454	6.9	3.4	0.08
Race							
White	88	75%		883	60%		0.004
Black	20	17.20%		460	31.60%		
Other	8	7.80%		111	8.40%		
Follow-up	116	17.7	10.0	1455	19.5	10.0	0.06
Diabetes	29	25.0%		1455	32.3%		0.12
Albumin(gm/dL)	116	3.6	0.4	1455	3.4	0.4	<0.0001
Serum Ferritin(ng/dL)	116	442.3	87.8	1455	594.4	13.5	0.005
Serum Iron(µg/dL)	115	82.4	25.5	1442	73.9	23.7	0.0003
TSAT(%)	98	28.5	14.2	1279	27.3	9.6	0.29
TIBC(µg/dL)	98	312.7	61.1	1287	274.7	56.1	<0.0001
Transferrin(mg/dL)	113	218.4	40.6	1441	192.1	38.3	<0.0001
K/tV	113	2.47	0.74	1425	2.25	0.57	0.0002
Renal	113	0.99	0.86	1425	0.55	0.68	<0.0001
Peritoneal	114	1.43	0.48	1447	1.68	0.52	<0.0001
PTH	116	284.9	146.9	1453	322.6	192.1	0.0395

*Standard Deviation of Hemoglobin.

# Coefficient of Hemoglobin Variability.

The group not requiring ESA had a greater proportion of males (69%). The proportion of sexes was equally distributed in the ESA group (53% males vs. 47% females). Since females may have menstrual blood losses which can partly account for variability in their hemoglobin, males were analyzed separately as well. By limiting the analysis to males only, the results did not change. The male no-ESA group still had a standard deviation of 1.0 gm/dL compared to 1.15 gm/dL in males who received ESA.

Patients in the no-ESA group had significantly more residual renal function, perhaps explaining the lack of EPO requirement in this group. The no-ESA group had slightly higher serum albumin and lower ferritins and PTH. Other iron indices (TIBC, Transferrin and total iron) were lower in the ESA treated group. A racial difference was noted in EPO requirement. A greater proportion of whites than blacks (9.1% vs. 4.2%) did not need ESA therapy to maintain similar levels of Hb. Separate subgroup analysis of the no-ESA group by race did not find any significant differences in Hb levels, iron indices, ratio of sexes, dialysis vintage or Kt/V. The black patients had significantly higher PTH overall- an effect that was consistent across the ESA and no-ESA groups.

**Figure 1:**
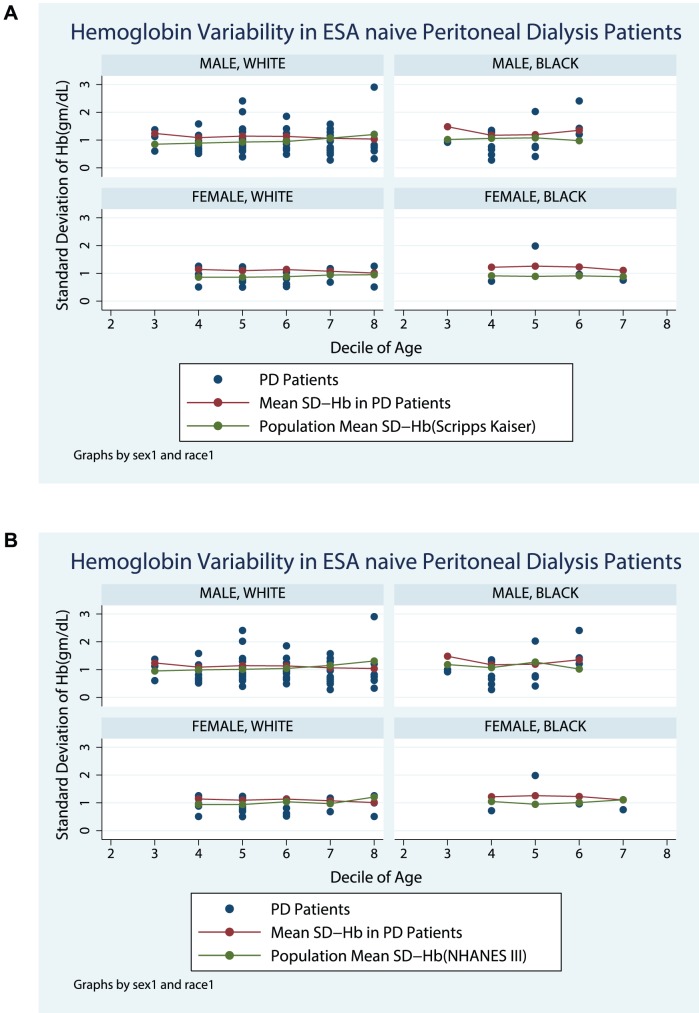
Hemoglobin Variability in ESA naïve Peritoneal Dialysis Patients.

**Figure 2:**
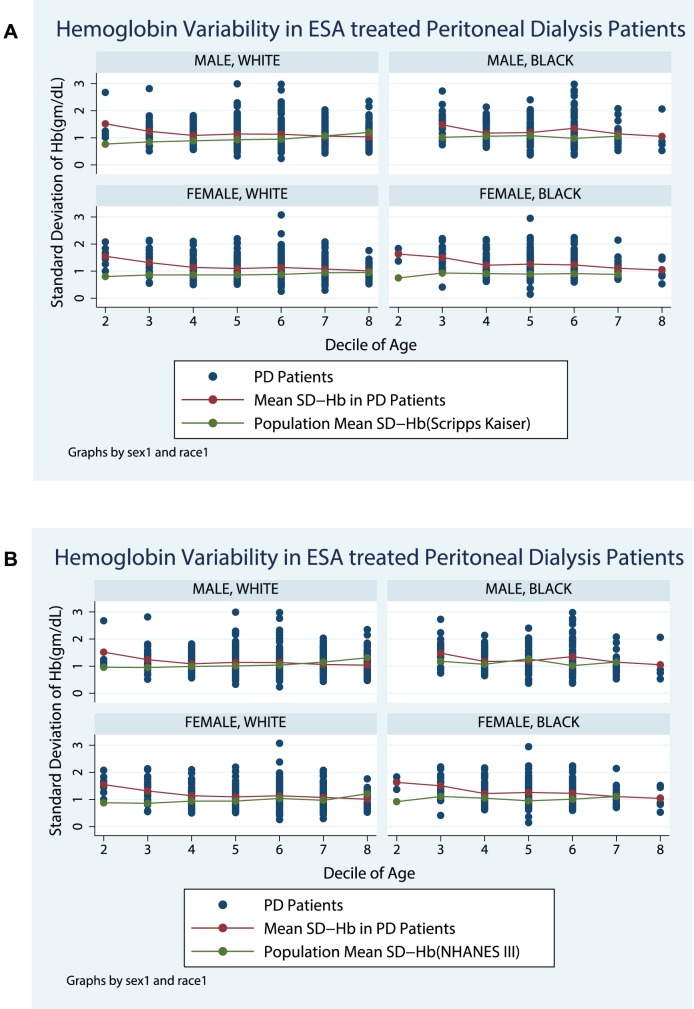
Hemoglobin Variability in ESA treated Peritoneal Dialysis Patients.

**Figure 3:**
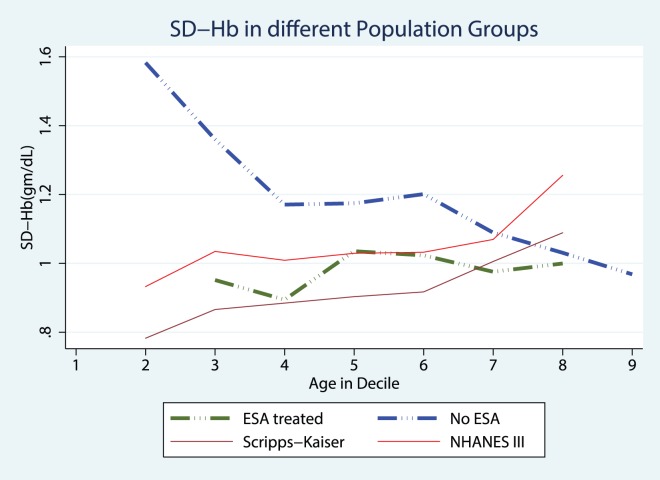
Hemoglobin Variability in different Population groups.

Figure 1a and 1b compare the SD-Hb seen in Peritoneal Dialysis not requiring any ESA therapy patients with population normals obtained from the Scripps-Kaiser database (Fig. 1a), and NHANESIII(Fig. 1b). Figure 2a and 2b compare the SD-Hb seen in ESA treated Peritoneal Dialysis patients with population normals obtained from the Scripps-Kaiser database (Fig. 2a), and NHANESIII (Fig. 2b). Hb-var in the ESA naïve group was similar in magnitude and trend as observed in the NHANESIII and Scripps-Kaiser populations (Fig. 3). In contrast the ESA treated group was distinctively different and the Hb-var was highest in younger patients. In older patients the Hb-var approached the population means and tended to decline in the patients with age more than 60 years. This was observed in both males and females as well as in Caucasians and African American patients.

Table 2 shows the results of a regression model with the p-values of significant predictors of SD-Hb in the ESA treated group. Age was again found to be a significant predictor of SD-Hb. SD-Hb declined by 0.05 gm/dL with each decile of age. Iron saturation and dialysis clearance (KT/V) were also found to be significant predictors of SD-Hb. Patient sex, race, vintage on dialysis, serum albumin, serum iron, transferrin and ferritin were not found to be significant predictors of SD-Hb. Using a stepwise regression model, in the ESA naïve group none of the factors studied were predictive of Hb-var.

**Table 2:** Stepwise regression model of SD-Hb.

SD-Hb	Coef.	Std. Err.	t	P>t	[95% Conf.	Interval]
Kt/V	−0.05859	0.031955	−1.83	0.067	−0.12139	0.004215
T-Sat	0.011898	0.001898	6.27	0	0.008168	0.015629
Age	−0.0048	0.001315	−3.65	0	−0.00738	−0.00221
Followup	−0.00454	0.00177	−2.56	0.011	−0.00802	−0.00106
Regression Constant	1.322492	0.132143	10.01	0	1.062788	1.582196

Patient Sex, Race, Vintage on Diaysis, Serum Albumin, Iron, Transferrin and Ferretin were removed at a threshold level of p = 0.1.

## Discussion

### Hemoglobin Variability - Normal or Abnormal

In the absence of a disease condition RBC production is controlled by a negative feedback system. The rate of change of RBC concentration is the difference between their rates of production and destruction. Within the bone marrow there exists a population of committed lineage specific pluripotent stem cells, also called colony forming units (CFU). CFUs are capable of differentiating into mature RBC under the influence of hormone erythropoietin produced by the kidneys. Low density of circulating RBC signals EPO production, whereas an increase in the numbers of circulating RBCs provides negative feedback to EPO. The pluripotent cells in the bone marrow exist in either a resting state (G_0_) or a proliferative state. In dialysis patients the effects of peripheral factors such as varying iron stores and circulating inflammatory mediators such as hepcidin [23] affecting bone marrow responsiveness to EPO have been studied extensively. It is not known, if these factors can also influence cell replication times within the marrow or change the rates of entry and exit of cells between the resting and proliferative states. Theoretically, hemoglobin variability in patients with CKD may originate from an altered rate of apoptosis of precursor cells within the proliferating compartment of the marrow(due to EPO deficiency), or it may occur due to an increased delay in cell maturation times within the marrow. Exogenous ESA administration may enhance (as is evident by a significantly greater Hb-var in the ESA treated group) or ameliorate this hemoglobin variability, but does not account for it entirely.

The existence of hemoglobin variability in the patients on dialysis not requiring ESA therapy provides useful insights into understanding RBC dynamics in CKD patients. Previous investigations have focused on the relationship between ESAs and Hb-var [6]–[11]; we think the Hb-var is inherent to the normal hematopoietic processes. In a review, Arneson et al. [24] have compared four different methodologies used to characterize hemoglobin variability. Using the mean, standard deviation or the absolute hemoglobin change [25], provides us with a summary measure that is easy to understand conceptually. A residual standard deviation methodology has been proposed by Feldman et al. It measures the deviation from a regression line and therefore controls for linear trends only in the data. Gilbertson et al. [8] have also used fluctuation across thresholds to characterize hemoglobin variability. These methods attempt to describe Hb-var but can be difficult to comprehend. They do not address the reasons for the hemoglobin variability and have not found clinical applications.

Peritoneal dialysis patients lack large volume shifts and do not have ongoing blood losses into dialysis tubings and dialyzers, which characterize hemodialysis which can affect Hb-var. Access related blood loss and use of hemodialysis catheters are also known to effect Hb-var [21] in hemodialysis patients but are not relevant to peritoneal dialysis patients. To test our hypothesis that Hb-var is present in chronic kidney disease independent of these factors, we chose to study patients on peritoneal dialysis. To exclude the effects of exogenous ESA on Hb-var, we studied patients who were not treated with any ESA. We found that patients on peritoneal dialysis with significantly preserved residual renal function still exhibited fluctuations in their Hb level. Hemoglobin variability (∼1 gm/dL) was present in ESA naïve patients and was significantly increased to 1.17 gm/dL by ESA therapy. This indicates that variability in Hb is inherent to disturbed RBC dynamics in CKD, independent of exogenous ESA administration.

### Determinants of Hemoglobin Variability

The inverse relationship between age and Hb-var in ESA treated hemodialysis patients has been observed previously [6], [21], but no teleological argument has been made to explain it. In our study of peritoneal dialysis patients, we found that the youngest patients had the maximum degree of variation in their Hb and also the maximum deviation from the population means. It can be hypothesized that the inflammatory cytokines and uremic milieu characteristic of chronic kidney disease disrupts the hemostatic processes that maintain Hb in a narrow range in healthy adults. Aging has been associated with a pro-inflammatory state and higher levels of IL-6, a key regulator of hepcidin [26], [27]. These similarities with CKD may explain the closer degree of resemblance between elderly ESA treated peritoneal dialysis patients and the apparently healthy elders in the general population. Studying the factors that affect cell replication and control movement of cells between the resting and actively proliferating marrow pools may help us devise new treatments for anemia in CKD patients. Mathematical models of RBC dynamics can help us travel the path from normal physiology to disease pathophysiology and ultimately, allow us to devise more rational therapies. The factors affecting Hb-var in patients not treated with ESA have not been investigated before. In our regression models none of the clinical parameters being studied were significant predictors of Hb-var in this subgroup of patients. This may be a result of relatively small number of patients, to have enough power to detect a difference. Indeed however, this is the first time a systematic study of patients not needing ESA has been undertaken. Larger analysis will be needed to confirm this finding.

### Determinants of Need for ESA therapy

Higher EPO requirements have been observed in African American patients on dialysis in previous observational studies [28], [29]. A race related genetic influence on hematopoiesis has been postulated earlier by Kauffman et al. [30]. Our finding that significantly more white patients did not need any ESA points to a stronger link to genetic factors effecting hematopoiesis. Similarly, greater proportion of males did not need ESA therapy. We did not have data related to hospitalizations, blood loss and infections which can also explain this apparent EPO hypo-responsiveness. The greater degree of residual renal function and total delivered dialysis dose (Kt/V) in the no-ESA group is not unexpected. The no-ESA group also had lower levels of various inflammatory markers including lower PTH, lower ferritin, and higher albumin level, once again pointing towards a role of inflammation in modulating erythropoiesis. Albumin levels have been found to be inversely correlated with inflammation in end stage renal disease patients [31]–[36]. Increased levels of cytokines INF-γ, TNF-α, IL-1 and IL-6 [37]–[39] have been implicated in producing ESA resistance and low hemoglobin levels. The low levels or absence of activation of these in patients not requiring ESA can be hypothesized but needs to be investigated. On the other hand ESA administration downregulates hepcidin [40], [41] and may also suppress cytokine levels [42], [43]. Lower levels hepcidin and pro-inflammatory cytokines may exert competing influence on erythropoiesis and can help explain the increased level of Hb-var in these patients. HFE gene mutations have been linked to lower ESA requirement [44], but their role in patients not needing ESA needs to be investigated. The prevalence of polymorphisms in genes for cytokine production can also provide useful insights. Compared to the study in hemodialysis patients [21] where inflammatory markers were significant predictors of Hb-var, serum albumin, PTH and ferritins were not found to be predictive of Hb-var in peritoneal dialysis patients. This is likely because peritoneal dialysis patients overall have lesser degree of inflammatory response compared to hemodialysis patients.

## Conclusions

We conclude that Hb-var cannot be attributed to exogenous ESA administration alone. The physiologic bases of Hb-var are not completely understood. Deregulated homeostatic mechanisms in chronic kidney disease may enhance Hb-var. Hb-var in peritoneal dialysis patients younger than 50 years of age may be 50% greater than in healthy adults. The normal biology of aging may determine Hb-var. Effects of aging such as increased levels of inflammatory mediators and diminished capacity to secrete erythropoietin may mimic chronic kidney disease. The interaction and effects of exogenous ESA administration on these factors remains poorly understood and should be investigated. Identification of these mechanisms may help us identify unique targets for novel therapies. By choosing appropriate physiologic targets for therapy we may be able to improve patient outcomes and enhance our understanding of disease processes.
